# Safety Helmet Detection Based on YOLOv5 Driven by Super-Resolution Reconstruction

**DOI:** 10.3390/s23041822

**Published:** 2023-02-06

**Authors:** Ju Han, Yicheng Liu, Zhipeng Li, Yan Liu, Bixiong Zhan

**Affiliations:** 1 China Construction First Group Construction & Development Co., Ltd., Beijing 100102, China; 2 College of Electrical Engineering, Sichuan University, Chengdu 610065, China

**Keywords:** deep learning, real-time detection, safety helmet detection, super-resolution reconstruction, YOLOv5

## Abstract

High-resolution image transmission is required in safety helmet detection problems in the construction industry, which makes it difficult for existing image detection methods to achieve high-speed detection. To overcome this problem, a novel super-resolution (SR) reconstruction module is designed to improve the resolution of images before the detection module. In the super-resolution reconstruction module, the multichannel attention mechanism module is used to improve the breadth of feature capture. Furthermore, a novel CSP (Cross Stage Partial) module of YOLO (You Only Look Once) v5 is presented to reduce information loss and gradient confusion. Experiments are performed to validate the proposed algorithm. The PSNR (peak signal-to-noise ratio) of the proposed module is 29.420, and the SSIM (structural similarity) reaches 0.855. These results show that the proposed model works well for safety helmet detection in construction industries.

## 1. Introduction

The construction industry is one of the most prone to safety accidents. Therefore, it is of great practical significance to study safety guarantees in this field. Over the past 20 years, it has experienced a decline in accident rates [1].

Head injuries can easily lead to a disability [2]. Reducing head injuries is the primary problem to ensure personnel security in the industry, and safety helmets are widely used to do so. The impact resistance of safety helmets can disperse the impact of rocks. Thus, many industrial regulations require workers to wear safety helmets during working. However, workers do not wear safety helmets as required due to the lack of safety awareness. Because of these reasons, safety accidents are frequent. According to relevant research statistics, 47.3% of people with head injuries in construction site accidents did not wear safety helmets [3]. Thus, it is very important to strengthen the supervision and management of workers. At present, the management of safety helmet wearing in most construction sites still requires manual monitoring. However, the efficiency of manual monitoring is very low due to the large working area and large flow of personnel. With the development of science and technology, video surveillance has become more and more popular. It is a vital part of safety helmet detection. Hence, the optimization of monitoring systems is widely studied [4,5]. Traditional video surveillance is mainly used for continuous monitoring. However, the final judgement still relies on humans’ decisions, and the degree of automation is not enough. Intelligence algorithms are a method to enhance automation. They are widely used in image processing, prediction, robotics and so on [6,7,8,9,10,11]. Currently, evolutionary algorithms and deep learning are two important intelligent systems [12,13,14]. Among them, deep learning is widely used in image processing because of its strong learning ability [15,16,17], which can be combined with video surveillance to solve the problems of traditional methods [18].

Slow transmission processes are caused by large image data collected by cameras. Therefore, the obtained original images need to be compressed before transmitting them so that the process can be accelerated. After compressed image data is transferred to the terminal main control unit, they are input into a YOLOv5 target detection network. Since the image compression usually reduces the image resolution, it can be restored by super-resolution (SR) reconstruction.

Many scholars apply deep-learning algorithms to the field of image SR reconstruction. Convolutional neural networks were first introduced in SR reconstruction [19]. Researchers added residual structure into the network to ensure convergence [20]. The attention mechanism structure has been added to further improve the performance of reconstruction [21]. However, in the detection of safety helmets, there exist the following problems to be solved. First, we use cameras to acquire images, and they are placed all over the construction sites. However, the tasks of image detection need computer terminals to complete, which are placed in the control room. Hence, image acquisition and detection are asynchronous in construction sites. Many studies have focused on detection; however, data transmission has not seen adequate research. In addition, previous popular datasets collected on the web have not considered construction sites, such as COCO128 and so on. In other words, the pictures in these datasets are taken from other environments. This problem leads to poor detection results of the model in construction sites.

To solve those problems, a safety helmet detection model is proposed, which is driven by an SR reconstruction network based on YOLOv5. To enhance the learning ability of the network, the double residual channel structure [22] is applied to an SR reconstruction network in the proposed model. PCAB and MPAB are introduced into the depth feature-extraction module of the main channel to improve the result of overall reconstruction. In summary, the contributions of this paper are as follows:

(1) A novel super-resolution (SR) reconstruction module is designed to improve the resolution of the image before the detection module. Compared with existing methods [23,24], this method reduces the influence of high-resolution image transmission on detection speed in the construction industry.
(2) A novel CSP (Cross Stage Partial) module of YOLO (You Only Look Once) v5 is presented to reduce the information loss and the gradient confusion.
(3) Based on the proposed SR reconstruction network and YOLOv5 network, a novel end-to-end safety helmet detection model is proposed to make the proposed model reach an average precision (AP) of 79.1%.
(4) More than 13,000 images are collected for safety helmet detection in construction sites.

The organization of the rest of this paper is as follows. Section 2 introduces the work related to target detection and SR reconstruction. Section 3 shows the structure and the details of the Dual-Channel Residual SR reconstruction model and the improved YOLOv5 model. The experimental details and results are given in Section 4. The conclusions are given in Section 5.

## 2. Related Work

Traditional safety helmet detection methods choose features manually for target detection. They have strong subjectivity, poor generalization ability and limitations in engineering applications. With the continuous development of deep-learning algorithms, researchers are applying deep-learning algorithms to target detection and image SR reconstruction.

### 2.1. Target Detection

At present, research on target detection algorithms include two-stage and one- stage algorithms. Two-stage detection algorithms generate a series of candidate boxes as samples and then classify samples through a convolutional neural network. This kind of detection method has higher task accuracy but slower speed. Girshick et al. [25] proposed the region convolutional neural network, fast regions with CNN [26] and faster regions with CNN [27] algorithms. A one-stage detection algorithm directly regresses the category probability and position coordinate values of objects through a backbone network without using a region proposal network (RPN). This kind of detection method sacrifices detection precision but improves detection speed. In 2016, Liu et al. [28] introduced the multiscaledetection method and proposed the SSD (single shot multibox detection) detection algorithm, which improved the detection accuracy. Redmon et al. [29,30,31] proposed YOLOv1, YOLOv2 and YOLOv3. The YOLOv1 network model abstracted the target detection task into a regression problem for the first time, which greatly sped up the target recognition speed. The YOLOv2 network model introduced a new basic model named darknet-19 based on YOLOv1 to realize end-to-end training. Compared with YOLOv1, the YOLOv2 network model realizes more accurate, faster and more target categories. YOLOv3 introduced the feature pyramid network (FPN) algorithm, promoted the new basic model darknet-53 and integrated three feature layers of different sizes for detection tasks. It improved detection speed and accuracy, especially the detection performance of small targets. Bochkovskiy et al. [32] proposed YOLOv4. This detection network takes CSP darknet-53 as the backbone network and uses the PANET path aggregation algorithm. As a result, it improved the detection accuracy of the model. In 2020, Jocher et al. [33] proposed YOLOv5. This network model adds a focus structure to the backbone network of YOLOv4 to obtain a balance between detection speed and accuracy. Carion et al. [23] proposed DETR for end-to-end object detection and brought transformers into the object detection fields. Recently, Wang et al. [34] proposed YOLOv7, which has achieved better accuracy and speed than YOLOv5.

### 2.2. SR Reconstruction

The image SR reconstruction algorithm is used to recover high-resolution images from one or more low-resolution images. Dong et al. [19] proposed SR Convolution Neural Networks (SRCNNs). SRCNNs effectively improve the results of image SR reconstruction compared with traditional image SR algorithms. However, the network is relatively simple, and the convergence speed is slow during the execution of the algorithm. In subsequent research, researchers added a residual structure to the convolution network to effectively solve the above problems. Kim et al. [23] proposed the VDSR network and increased the number of layers of the CNN to 20. The residual structure and CNN are embedded into image SR reconstruction, and the image reconstruction result is improved. Li et al. [20] proposed a multiscale residual network (MSRN). This network includes image multiscale features in the residual structure to further improve the image reconstruction result. Zhang et al. [35] proposed the residual channel attention network SRCAN. This network applies a channel attention mechanism to the image SR problem and achieves a better reconstruction effect than previous algorithms. Lu et al. [36] presented a novel recursive unit for SR reconstruction fields to force models to learn more details by learnable up-sampling methods. Liu et al. [37] proposed an attention-based approach to discriminate between texture areas and smooth areas.

## 3. Materials and Methods

**Figure 1:**
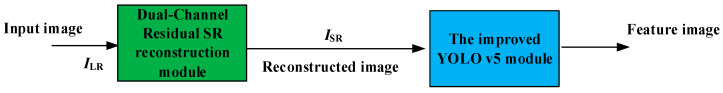
The overall architecture of the proposed module.

In this paper, the proposed safety helmet detection model is designed based on a Dual-Channel Residual SR reconstruction module and an improved YOLOv5 module. The overall architecture is given in Figure 1. *I_LR_* means the input image features and *I_SR_* means the reconstructed image features. The two submodules in this figure are addressed as follows.

### 3.1. Dual-Channel Residual SR Reconstruction Module

**Figure 2:**
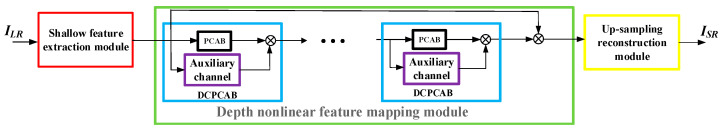
The structure of the Dual-Channel Residual SR reconstruction module.

The SR reconstruction module consists of three modules: a shallow feature extraction module, a depth nonlinear feature mapping module and an up-sampling reconstruction module. The specific structure is shown in Figure 2.

The shallow feature-extraction module is an ordinary convolution layer. As shown in Figure 2, the shallow extracted feature *F_L_* is obtained from the input original low-resolution image *I_LR_* through this module.

**Figure 3:**
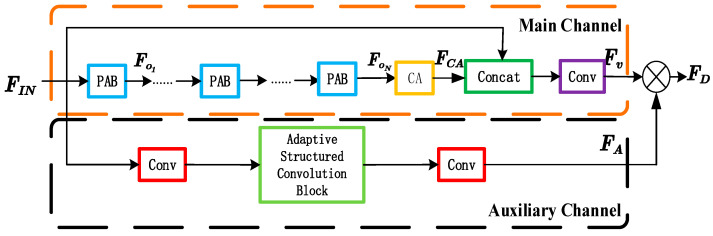
The DCPCAB architecture.

The depth nonlinear feature-mapping module is composed of several dual-channel pixel-channel attention blocks (DCPCABs). Each DCPCAB is shown in Figure 3. In this figure, the main channel of the DCPCAB module is composed of several pixel attention blocks (PABs), a channel attention (CA) block and a convolution layer. In this paper, the number of PABs in DCPCAB is two. The auxiliary channel is composed of two convolution layers and an adaptive structured convolution block.

**Figure 4:**
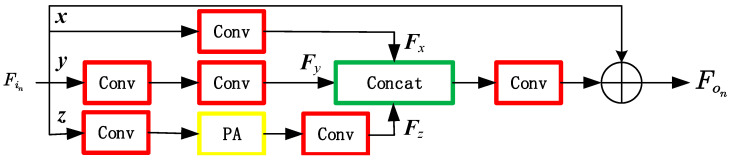
The PAB architecture.

The architecture of PAB is shown in Figure 4. In this figure, *F_in_* is the input feature, and it is put into three branches *x*, *y* and *z*. One convolution layer with a 1 × 1 kernel size is adopted in branch *x* to reduce the input feature *F_in_* to the output feature *Fx*. In branch *y*, the input features are first fed into a convolution layer with a 1 × 1 kernel size for dimension reduction and then put into a convolution layer with a 3 × 3 kernel size for feature extraction to obtain the output feature *Fy*. In branch *z*, the input feature is first fed into a convolution layer for dimension reduction. The reduced dimension feature is input into the pixel attention (PA) mechanism network for pixel-level feature weighting. A convolution layer is adopted for feature extraction to obtain the output feature given as

(1) $$F_{z}=conv_{3\times 3}\left(F_{PA}\left(conv_{1\times 1}\left(F_{in}\right)\right)\right)$$

where $conv_{3\times 3}$ represents the convolution operation with a 3 × 3 kernel size. *F_PA_* can be given by

(2) $$F_{PA}\left(conv\left(F_{in}\right)\right)=conv_{1\times 1}\left(conv_{1\times 1}\left(F_{in}\right)\right)*\delta \left(conv_{1\times 1}\left(conv_{1\times 1}\left(F_{in}\right)\right)\right)$$

where *δ* is the sigmoid activation function.

The output features of the three branches are combined through a *concat* operation given by

(3) $$F_{o}=conv_{1\times 1}\left(concat\left(F_{x},F_{y},F_{z}\right)\right)+F_{in}$$

where *concat* means the operation of channel merging, which is used to merge the three features.

There are multiple PAB blocks in a DCPCAB module in Figure 3. The output *F_on_* of each PAB can be iteratively calculated as

(4) $$F_{on}=conv_{1\times 1}\left(concat\left(F_{xn}+F_{yn}+F_{zn}\right)\right)+F_{o\left(n-1\right)}$$

*F_CA_* in Figure 3 is the CA output and is obtained by Equation (5). The output of **CA** can be given by

(5) $$F_{CA}=conv_{1\times 1}\left(F_{on}\right)⊗sigmoid\left(conv_{1\times 1}\left(F_{GAP}\left(F_{on}\right)\right)\right)$$

where FGAP stands for the global average pooling operation and ⊗ means the pointwise multiplication operation.

The main channel output *Fv* in Figure 3 can be obtained by

(6) $$F_{v}=conv_{1\times 1}\left(concat\left(F_{CA}+F_{IN}\right)\right)$$

where *F_IN_* is the input of the DCPCAB.

The SR reconstruction operation always makes the edge information of the original images blurred or even deformed. The auxiliary channel module is introduced to broaden the width of the whole network to solve these problems. Adaptive structured convolution blocks are added in the auxiliary channel. The modules are adaptive to different expansion rates according to different image sizes. They can make the whole depth nonlinear feature-mapping submodule focus on the extraction of high-frequency features of the image. The operation of the auxiliary channel is given by

(7) $$F_{A}=conv\left(F_{DC}\left(conv\left(F_{IN}\right),rate\right)\right)$$

where *F_DC_* refers to the expansion convolution operation and *rate* refers to the expansion rate.

The final output FD in Figure 3 is given by

(8) $$F_{D}=F_{A}⊗F_{v}$$

The output of module *Fout* in Figure 2 can be calculated by

(9) $$F_{out}=conv\left(F_{DN}\right)+F_{L}$$

where *F_DN_* means the output of the final DCPCAB.

The up-sampling reconstruction module in Figure 2 can be given by

(10) $$I_{SR}=conv\left(F_{up}\left(F_{out}\right)\right)$$

where *F_up_* represents the up-sampling operation and *I_SR_* represents the results of the up-sampling reconstruction module.

To optimize the proposed **SR** reconstruction network, a loss function is adopted as

(11) $$L1=\frac{1}{k}\overset{k}{\underset{i=1}{\sum }}{\left|\left|I_{SR}-I_{HR}\right|\right|}_{1}$$

where ${\left|\left|\;\right|\right|}_{1}$ means an *L*1 norm, *k* represents the number of training pictures and *I_HR_* represents the corresponding high-resolution image of the *I_SR_*. In this paper, the final loss of the best results is 0.0034, and the meaning of it is the MAE of the resolution of the reconstructed image and high resolution image.

The receptive field and the speed of the SR reconstruction models can be improved by the dual-channel residual structure. The number of parameters in the SR reconstruction model can be reduced by the PCAB structure. In other words, the PCAB structure can make the model more lightweight.

***Remark 1***: Here, the proposed Dual-Channel Residual SR reconstruction model is compared with SRCNN and SRGAN. Neither of these models consider the receptive field or the speed of the SR reconstruction model. SRCNN effectively improves the results of image SR reconstruction compared with traditional image SR algorithms. However, the network is relatively simple, and the convergence speed is slow during the execution of the algorithm. SRGAN [38] considers restoring fine-grained texture details. To improve the receptive field and the speed of the SR reconstruction model, the dual-channel is used in the proposed model. The number of parameters in the SR reconstruction model can be reduced by the PCAB structure. When other architectures were used instead of the dual-channel structure, the number of parameters we need to train must be the product of multiple dimensions. But the dual-channel can halve the number of parameters by introducing dual channels. In other words, the PCAB structure can make the model more lightweight.

### 3.2. The Improved YOLOv5 Module

YOLOv5 is an improved version algorithm based on YOLOv4 proposed by the Ultralytics LLC company. It is a network with excellent detection accuracy and speed in a single-stage detection network. YOLOv5 has a good detection effect on Pascal visual object classes (Pascal VOC) and common objects in context (COCO) target detection tasks, so YOLOv5 is selected as the detection network.

**Figure 5:**
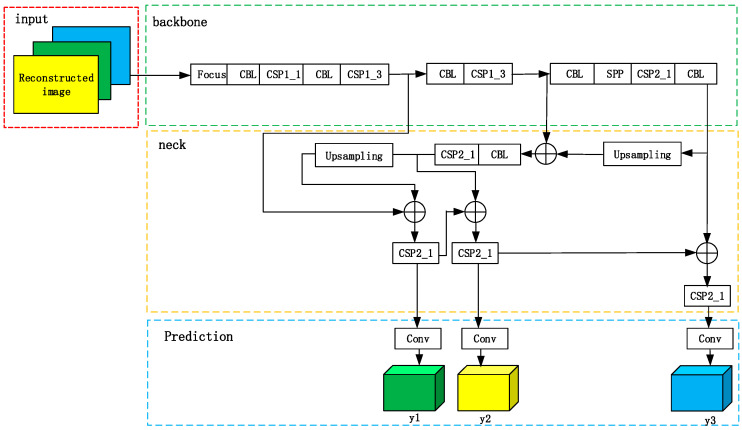
The structure of the improved YOLOv5 module.

The YOLOv5 network structure is divided into four parts: the input port, backbone network, neck part and prediction part. The structure is shown in Figure 5 [33].

**Figure 6:**
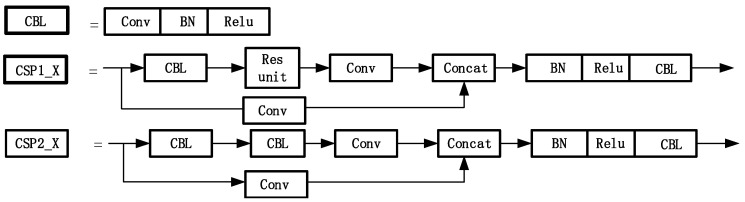
The structures of CBL, CSP1_X and CSP2_X.

The input port is used to mosaic random images to enrich the datasets, calculate the adaptive anchor frame and zoom images adaptively. The backbone mainly adopts a focus structure and cross-stage partial (CSP) structure to obtain features. The focus in the backbone is used to slice the input image data. The structure combining three multiscale pooling layers is used to improve the receptive field of the network while minimizing the loss of speed. It is helpful for the network to extract the important image features, reduce the image loss caused by early image processing and further improve the detection accuracy of the model. The structures of CSP and CBL are shown in Figure 6.

**Figure 7:**
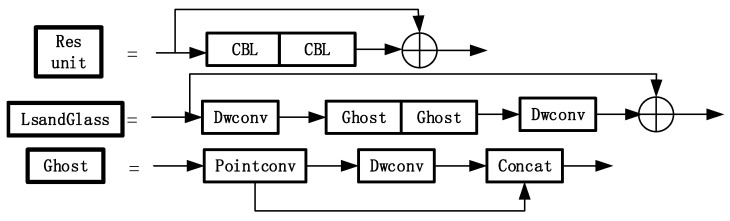
The structure of the Res unit, LSandGlass and Ghost.

The CSP1_*X* module is improved in two parts in Figure 7. The original CSP structure of YOLOv5 can lead to problems such as information loss and gradient confusion. Therefore, we use the LSandGlass module to replace the Res unit residual module in YOLOv5 and the 3 × 3 depth space convolution layer. The LSandGlass is different from the bottleneck structure with deep spatial convolution in China construction, 3 × 3 deep space convolution Dwise layers are moved to both ends of the residual path with high dimensional representation and the CBL blocks are stated in the mid. Two-deep convolution can encode more spatial information and make more gradients propagate across multiple layers, thus reducing information loss.

Dwconv is moved to both ends of the residual path with high-dimensional representation to realize gradient propagation across multiple layers and reduce the loss of information. Considering such processing can increase the overall computation, the Ghost module is used to replace the CBL module of the bottleneck module in CSP. This scheme is adopted to reduce the computation and to compress the model size compared with the original 3 × 3 standard convolution.

The neck module of YOLOv5 uses the structure of feature pyramid networks (FPN) and pyramid attention networks (PAN). The prediction module contains the bounding box loss function and the non-maximum suppression (NMS) function. YOLOv5 uses the binary cross entropy loss function to calculate the loss of category probability and target confidence score. In the experiment, CIOU loss is selected as the bounding box loss function. The related formulas are given as [39]

(12) $$CIOU\;Loss=1-\left(IOU-\frac{d_{1}{}^{2}}{d_{2}{}^{2}}-\beta \theta \right)$$

(13) $$\beta =\frac{\theta }{\left(1-IOU\right)+\theta }$$

(14) $$\theta =\frac{4}{\pi^{2}}{\left(tan^{-1}\frac{W^{g}}{h^{g}}-tan^{-1}\frac{W}{h}\right)}^{2}$$

where *d*_1_ represents the Euclidean distance between the prediction box and the centre point of the target box and *d*_2_ represents the diagonal distance of the minimum circumscribed matrix. $\frac{W^{g}}{h^{g}}$ represents the aspect ratio of the target frame, and $\frac{W}{h}$ represents the aspect ratio of the predicted frame.

***Remark 2*:** Here, the improved YOLOv5 model is compared with the original YOLOv5 model. The original YOLOv5 model did not consider problems such as information loss and gradient confusion. The proposed YOLOv5 model uses the LSandGlass module to replace the Res unit residual module in YOLOv5 to solve these problems. Considering that such processing can increase the overall computation, the Ghost module is used to replace the CBL module to solve it.

## 4. Results

### 4.1. Experimental Setup

First, we collected the image datasets by ourselves, which all come from construction sites and depict safety helmets. We first obtained the videos from construction sites and then used the VOTT to get images from the videos. The time of each video is about 14s, and we cropped at 7 frames per second to obtain the experimental images. The number of the images in the datasets is about 13,000, and the resolution of each image is 610 × 480. Then, we used pure interpolation to resize the input images to get 2× low-resolution.

To realize fast and reliable results, the entire method was implemented on a workstation equipped with two NVIDIA TITANRTX GPUs and an Intel i9 CPU.

All coding work was based on Python 3.7 and PyTorch 1.7.

The initial learning rates of the SR and detection were 0.0001 and 0.01, respectively.

The training epoch times of SR and detection were all 100.

The prediction times of SR and detection were all 100.

The kernels of the SRCNN were 1 × 1, 5 × 5 and 9 × 9.

The number of residual block layers for the generator in SRGAN was 16, and the weights of the loss function for SRGAN was given as 1, 1 and 1.

The Adam optimizer was applied with a momentum of 0.9, and the batch size was 32.

The training and test datasets were collected by CSCEC-2020Z-10.

### 4.2. Metrics

The structural similarity values (SSIM) and the peak signal-to-noise ratio (PSNR) are used to measure the quality of the reconstructed images. Among them, the former is adopted to measure the difference between the original image and the SR reconstructed image. The latter is used to measure the difference between the original image and the SR reconstructed image.

**PSNR** is given by

(15) $$PSNR=10log_{10}\frac{255}{\frac{1}{3}\sum_{}{\left(MSE\right)}_{C}}$$

where **MSE** is the indicator of the square error for the image.

**SSIM** is defined as

(16) $$SSIM\left(I_{0},I_{1}\right)=\frac{\left(2m_{0}m_{1}+c_{1}\right)\left(2\sigma_{0}\sigma_{1}+c_{2}\right)}{\left(m_{0}^{2}+m_{1}^{2}+c_{1}\right)\left(\sigma_{0}^{2}+\sigma_{1}^{2}+c_{2}\right)}$$

where $I_{0}$ and $I_{1}$ are the original and the reconstructed high-resolution images. *m* is the indicator of the mean, and σ is the variance. $c_{1}$ and $c_{2}$ are both constants. In this paper, $c_{1}$ is set as $0.01\times 255^{2},$ and $c_{2}$ is set as $0.03\times 255^{2}$.

Precision (**P**), Recall (**R**) and Average Precision (**AP**) are used to measure the detection tasks. Among them, Precision is used to describe the ratio of predicted positive examples to all positive examples, and it is calculated by

(17) $$P=\frac{TP}{TP+FP}\times 100\%$$

where *P*, *TP* and *FP* indicate the precision, true positives and false positives, respectively.

Recall is used to describe how many of the positive samples were detected in the prediction, and it is given by

(18) $$R=\frac{TP}{TP+FN}\times 100\%$$

where *FN* indicates false negatives.

Average Precision synthesizes *P* and *R*, which is calculated from the area under the precision–recall curve and can be given by

(19) $$AP=\frac{TP+TN}{TP+FP+TN+FN}\times 100\%$$

where *TN* indicates true negatives.

### 4.3. SR Reconstruction Experiments

**Figure 8:**
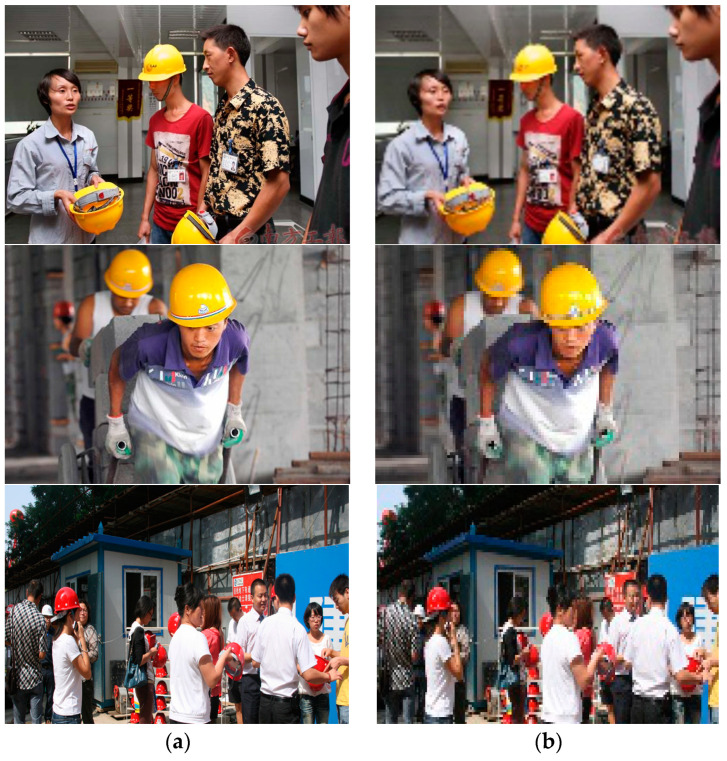
Examples of input images in the SR reconstruction module. (**a**) The output of the training datasets; (**b**) the input of the training datasets.

The SR reconstruction experiments are trained on approximately 80% of the 13,000 original images. They are validated on about 10% of the images and tested on the remaining 10%. Examples of the input images are shown in Figure 8. The right three subfigures show the low-resolution input images, and the left three subfigures are the output images.

**Figure 9:**
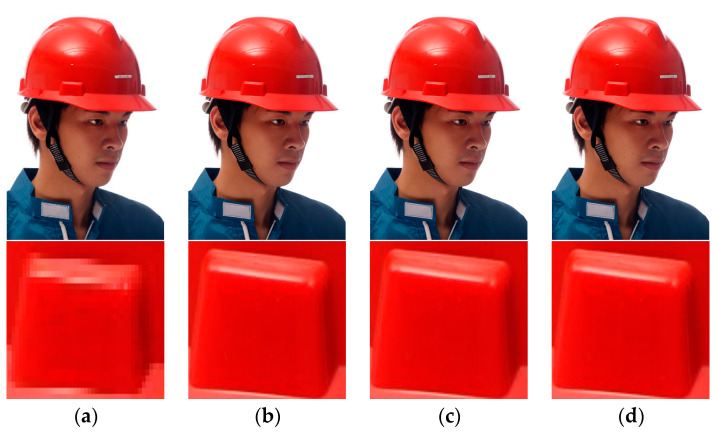
Examples of the results. (**a**) The input of the SR reconstruction module; (**b**) the output of SRCNN; (**c**) the output of SRGAN; (**d**) the output of the Dual-Channel Residual SR reconstruction module.

The different super-resolution reconstruction structures are shown in Table 1, and the experiments’ results are shown in Table 2. Examples of the results are shown in Figure 9. It can be observed that the PSNR of the proposed method improved by 16.22% compared with SRCNN and by 5.36% compared with SRGAN. This means that the reconstructed images using the proposed method are closer to the original images. The SSIM of the proposed method improved by 9.76% compared with SRCNN and by 4.27% compared with SRGAN. These results mean that the proposed method can extract more image-structural information for human eyes.

### 4.4. Safety Helmet Detection Experiments

To verify the advantage of the proposed model in safety helmet detection, we compare the Precision, Recall and AP with those of other models. Each model employs the improved YOLOv5 and the original YOLOv5. The datasets are trained by different SR reconstruction methods first and different YOLO methods second.

From Table 3, it can be observed that the proposed model obtains a larger value of Precision compared with other models. This means that the proposed model has a better ability to identify safety helmets. It can also be observed that the proposed model obtains the largest values of Recall compared with the other models. This means the proposed model’s ability to find all the safety helmets is the best. The AP value of the proposed model compared with SRCNN+YOLOv5 improved by 25.96% and by 11.10% compared with SRGAN+YOLOv5. Furthermore, when both use the Dual-Channel Residual SR reconstruction module, the AP value of the improved YOLOv5 is approximately 0.64% higher than that of YOLOv5. It is obvious that the LSandGlass module can realize better detection results than the res module. Moreover, the original YOLOv5 is more affected by image resolution. The proposed SR reconstruction module with improved YOLOv5 improved by 23.07% compared with SRCNN with improved YOLOv5 and by 9.25% compared with SRGAN with improved YOLOv5. The proposed SR reconstruction module with the original YOLOv5 improved by 25.16% compared with SRCNN with the original YOLOv5 and by 10.39% compared with SRGAN with the original YOLOv5. These results show that the improved YOLOv5 has better robustness in detection tasks.

**Figure 10:**
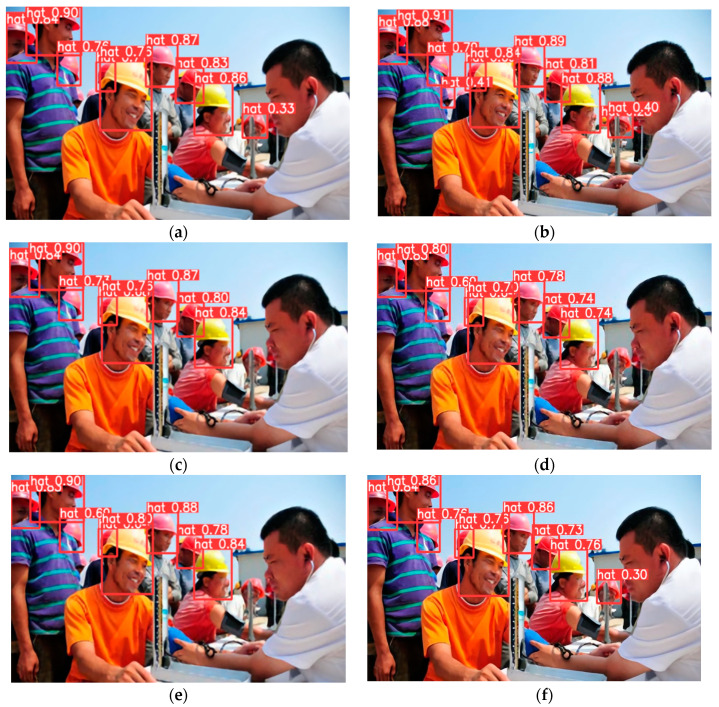
Target detection results obtained using different methods. (**a**) The result obtained using the proposed SR resolution module with the original YOLOv5 network; (**b**) the result obtained using the proposed SR resolution module with the improved YOLOv5 network; (**c**) the result obtained using SRGAN with the original YOLOv5 network; (**d**) the result obtained using SRGAN with the improved YOLOv5 network; (**e**) the result obtained using SRCNN with the original YOLOv5 network; and (**f**) the result obtained using SRCNN with the improved YOLOv5 network.

As shown in Figure 10, when the features of safety helmets are obvious in the image, the proposed model has very good recognition of the safety helmets. Comparing (b) with (d) and (f), these results are obtained by the same detection method and different SR reconstruction methods. The number of valid detection boxes in (b) is much greater than that in (d) and (f). The Precision in (b) is also larger than those in (d) and (f). These results mean that the proposed SR reconstruction method obtains better performance than SRCNN and SRGAN. Comparing (a) with (b), (c) and (d), (e) and (f), the valid detection boxes in (b), (d) and (f) are more than those in (a), (c) and (e). The above results show that the improved YOLOv5 achieves a higher recognition ratio of safety helmets; furthermore, the proposed method can obtain more accurate positioning and higher recognition precision for safety helmet detection. This means that the improved YOLOv5 is superior to the original YOLOv5. The above results indicate that the proposed model can achieve better detection results than other models.

## 5. Discussion and Conclusions

A novel safety helmet detection model is presented to implement super-resolution reconstruction-driven safety helmet detection. At construction sites, the images collected need to be transmitted to the terminal for detection. The resolution of images is lowered to make it faster. This can lead to a reduction in the detection accuracy. A novel detection model is proposed to overcome this problem. It consists of two modules. First, the SR reconstruction module is used to improve the image quality. Then, to finish the helmet detection, a novel YOLOv5 module is used as the detection module. They are trained separately but tested by the proposed datasets together. The experimental results show that the proposed SR module can increase the PSNR value while maintaining a consistent SSIM value compared with some existing SR reconstruction methods. It demonstrates the superiority of the proposed model. Based on the current results, the proposed model is a feasible tool for safety helmet detection. It can be easily used in construction monitoring or traffic safety monitoring. This paper mainly uses the individual models on specific tasks and combines the models to achieve the whole task. In the future, we will continue to realize the integrated design of SR reconstruction and YOLOv5 to reduce design redundancy. At the same time, we will implement a lightweight model and improve the computational effectiveness. Besides that, we will consider the noise in images coming from industrial sites in the future research.

## Figures and Tables

**Table 1:** The difference of the three SR reconstruction methods.

Model	Structure
**SRCNN [19]**	Three parts: input, non-linear mapping and output
**SRGAN [35]**	Two parts: generator network and discriminator network
**Dual-Channel Residual SR Reconstruction model**	Three parts: input, Dual-channel module and output

**Table 2:** The results achieved by different SR reconstruction methods.

	Model	SRCNN [19]	SRGAN [35]	Dual-Channel Residual SR Reconstruction Model
Metrics				
**PSNR**		25.313	27.923	29.420
**SSIM**		0.779	0.820	0.855
**Parameters’ number**		7,235,377	73,478,945	3,525,431

**Table 3:** Comparison with other models in safety helmet detection.

	Model	Precision (%)	Recall (%)	AP (%)
Metrics				
**SRCNN [19]+YOLOv5**		73.2	55.1	62.8
**SRCNN+improved YOLOv5**		74.1	58.3	64.3
**SRGAN [35]+YOLOv5**		81.3	61.1	71.2
**SRGAN+improved YOLOv5**		84.3	60.3	72.4
**Dual-Channel Residual SR reconstruction module+YOLOv5**		88.4	71.5	78.6
**Dual-Channel Residual SR reconstruction module+improved YOLOv5**		88.6	71.5	79.1
